# The clinical characteristics, novel predictive tool, and risk classification system for primary Ewing sarcoma patients that underwent chemotherapy: A large population‐based retrospective cohort study

**DOI:** 10.1002/cam4.5379

**Published:** 2022-10-21

**Authors:** Chao Huang, Qiu‐Ping Yu, Zichuan Ding, Zongke Zhou, Xiaojun Shi

**Affiliations:** ^1^ Department of Orthopedics West China Hospital of Sichuan University Chengdu China; ^2^ Health Management Center West China Hospital of Sichuan University Chengdu China

**Keywords:** cancer‐specific survival, chemotherapy, Ewing sarcoma, nomogram, risk stratification system, SEER

## Abstract

**Background:**

This study aims to determine the independent prognostic predictors of cancer‐specific survival (CSS) in patients with primary Ewing sarcoma (ES) that underwent chemotherapy and create a novel prognostic nomogram and risk stratification system.

**Methods:**

Demographic and clinicopathologic characteristics related to patients with primary ES that underwent chemotherapy between 2000 and 2018 were extracted from the Surveillance, Epidemiology, and End Results (SEER) database. CSS was the primary endpoint of this study. First, independent prognostic predictors of CSS identified from univariate and multivariate Cox regression analyses were used to construct a prognostic nomogram for predicting 1‐, 3‐, and 5‐year CSS of patients with primary ES that underwent chemotherapy. Then, calibration curves and receiver operating characteristic (ROC) curves were used to evaluate the nomogram's prediction accuracy, while decision curve analysis (DCA) was used to evaluate the nomogram's clinical utility. Finally, a mortality risk stratification system was constructed for this subpopulation.

**Results:**

A total of 393 patients were included in this study. Age, tumor size, bone metastasis, and surgery were independent prognostic predictors of CSS. The calibration curves, ROC, and DCA showed that the nomogram had excellent discrimination and clinical value, with the 1‐, 3‐, and 5‐year AUCs higher than 0.700. Moreover, the mortality risk stratification system could effectively divide all patients into three risk subgroups and achieve targeted patient management.

**Conclusions:**

Based on the SEER database, a novel prognostic nomogram for predicting 1‐, 3‐, and 5‐ year CSS in patients with primary ES that underwent chemotherapy has been constructed and validated. The nomogram showed relatively good performance, which could be used in clinical practice to assist clinicians in individualized treatment strategies.

## Graphical abstract

Three hundred and ninety three patients with primary Ewing sarcoma (ES) that underwent chemotherapy were collected from the SEER database. Age, tumor size, bone metastasis, and surgery were identified as independent prognostic factors for the cancer‐specific survival (CSS) of patients with primary ES that underwent chemotherapy. Nomograms for predicting 1‐, 3‐, and 5‐year CSS for patients with primary ES that underwent chemotherapy were constructed based on identified prognostic factors. The mortality risk classification system based on the nomogram could effectively divide patients with primary ES that underwent chemotherapy into three risk subgroups.

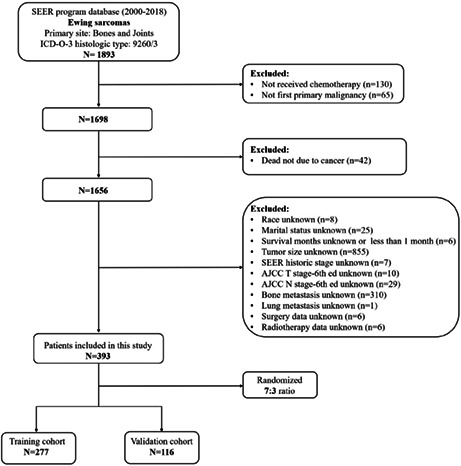

## 1 INTRODUCTION

Ewing sarcoma (ES) was first reported by James Ewing in 1921, describing it as a primary, separate, malignant tumor with no osteogenic properties.^1^ ES is now considered a highly malignant sarcoma of bone or soft tissue originating from the neuroectoderm.^2^ It is the second most common primary malignant tumor of bone in adolescents after osteosarcoma, accounting for 9.17% of malignant bone tumors. In the United States, the overall incidence of ES is about 2.93 cases/1,000,000.^3^ Due to its highly malignant nature, it has become a worldwide health challenge, affecting skeletal growth in adolescents and even causing disability and deformity. Currently, the treatment for ES includes surgery, chemotherapy, and radiotherapy. Among them, chemotherapy is the standard of initial treatment, and more than 90% of ES patients received chemotherapy reported in the literature.^4^, ^5^, ^6^ Chemotherapy is effective for local, multiple, and metastatic ES, reducing the amputation and recurrence rate and improving the survival rate. Before applying chemotherapy, metastases appeared rapidly in ES patients, making local surgical treatment equivalent to a palliative treatment method. Most patients would soon die due to metastatic disease. As a result, the early reported survival rates of ES were less than 10%.^2^, ^7^ However, with the continuous development of chemotherapy, the 5‐year overall survival (OS) of ES patients has improved from 25% to nearly 60% after adequate local tumor resection combined with chemotherapy. A 5‐year survival rate of 65% could be achieved for non‐metastasis ES patients that underwent chemotherapy.^8^

Studies focused on exploring the factors affecting the survival of primary ES patients treated with chemotherapy have been reported. Gaetano et al. concluded that sex, age, tumor volume, serum lactate dehydrogenase (LDH) level, type of local treatment, chemotherapy regimen, and histological response to preoperative treatment were significantly associated with 5‐year disease‐free survival of patients with non‐ metastasis ES patients that underwent chemotherapy.^9^ Another study showed that male, age > 12 years, fever, anemia, high LDH levels, and tumor located outside the extremities were associated with a high risk of recurrence in non‐metastasis ES patients that underwent chemotherapy.^10^ Meanwhile, they suggested that all of these variables should be included in the criteria to categorize patients according to the risk of recurrence. However, it is not easy to achieve fast and effective survival prediction in the face of so many variables. The Enneking staging system for benign and malignant tumors of the musculoskeletal system and the American Joint Committee on Cancer (AJCC) staging system for soft tissue sarcoma are widely used in clinical practice and patient management based on multivariate formation, but neither can accurately predict the survival of patients.

Nomogram is a visual statistical prediction tool to identify clinical disease‐related prognostic factors, and it has been widely used in various cancers, including breast, lung, and thyroid cancer.^11^ By integrating various critical prognostic factors, the nomogram predicts and quantifies the survival rate of individual tumor patients. However, no nomogram has been developed to predict survival in primary ES patients treated with chemotherapy. There is a need to re‐examine these patients to identify the variables closely associated with survival to assist clinicians in improving patient counseling, better risk stratification, “tailoring” follow‐up monitoring, and guiding appropriate treatment for those patients.^12^ Meanwhile, in the treatment and management of cancer patients, the ultimate goal is to improve patient survival. OS and cancer‐specific survival (CSS) are commonly used, but CSS can provide a closer relationship with tumor‐mediated patient prognosis than OS and provide more precise guidance for the subsequent analysis of these patients. Therefore, we chose CSS as the primary endpoint. This study aimed to identify relevant independent prognostic predictors of CSS in primary ES patients treated with chemotherapy by assessing relevant data from the Surveillance, Epidemiology and End Results (SEER) database to develop a novel nomogram and risk stratification system for predicting 1‐, 3‐ and 5‐year CSS probability of primary ES patients treated with chemotherapy and enabling stratified management of patients with different mortality risks.

## 2 METHODS

### 2.1 Database

All the data in this study were collected from SEER database (https://seer.cancer.gov/seerstat/), which usually collected data from 18 population‐based cancer registries covering approximately 30% of the US population.^13^ The data of this subpopulation in the present research were downloaded from SEER database using SEER Stat 8.3.9.2 software with the reference number 16336‐Nov2020 (Incidence‐SEER Research Plus Data, 18 Registries, Nov 2020 Sub [2000–2018]). SEER is a publicly available database, and the acquired data does not disclose specific personal information; thus, the ethics committee's approval or the patient's informed consent do not require. This research was conducted following the Strengthening the Reporting of Observational Studies in Epidemiology (STROBE) guidelines.^14^

### 2.2 Population selection

**FIGURE 1:**
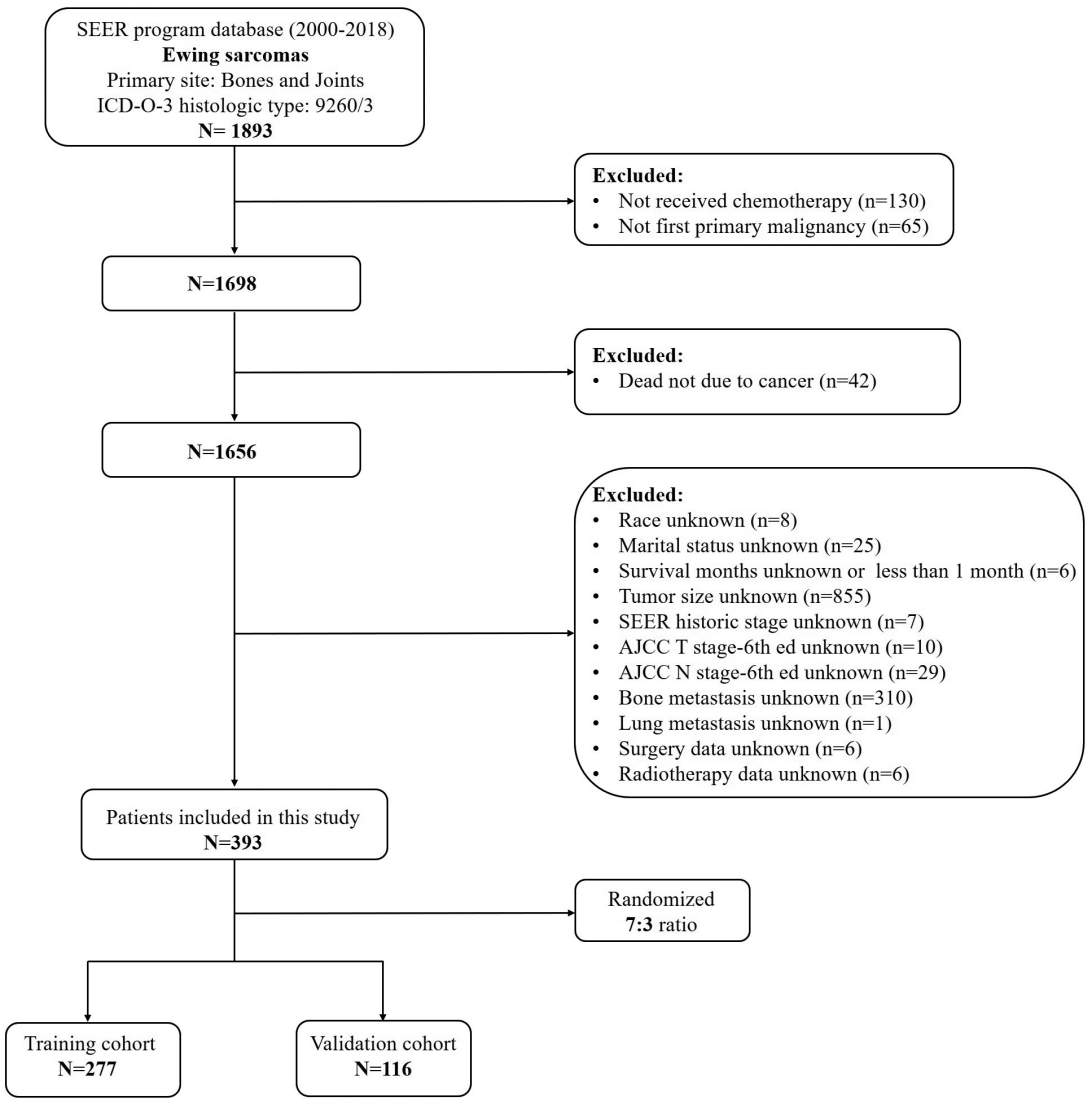
The flowchart for patient selection in this study.

The inclusion criteria included: (1) The site recode ICD‐O‐3/WHO 2008 was Bones and Joints; (2) 9260/3: ICD‐O‐3 histology code; (3) received chemotherapy; (4) primary tumor; (5) complete follow‐up data. The exclusion criteria were as follows: (1) survival months less than 1 month; (2) multiple primary cancer; (3) the information about age, sex, race, marital status, Derived AJCC T (T stage), Derived AJCC N (N stage), SEER historic stage A (tumor stage), surgery, radiotherapy, tumor size, and distant (bone, brain, liver, and lung) metastases were unknown. Finally, 393 patients were included in this study (Figure 1). They were randomly divided into a training cohort and a validation cohort according to a ratio of 7:3. We used the training cohort to identify the CSS‐related independent prognostic predictors and constructed a prognostic nomogram and risk stratification system for that subpopulation, which was verified by the validation cohort.

### 2.3 Variable definitions

We selected patients' demographic characteristics (age, sex, race, marital status, and survival time), tumor characteristics (tumor size, primary site, T/N stage, and tumor stage), distant (bone, brain, liver, and lung) metastases, and treatment information (surgery and radiotherapy) to analyze. According to X‐tile software, the optimal cut‐off values for age and tumor size were 13 and 28 years old, 54 and 135 mm, respectively (File S1).^15^ Then tumor size was categorized into three groups, <54, 54–135, and > 135 mm, while the age was divided into <13, 13–28, and > 28 years old, respectively. Sex was divided into male and female, and the race was divided into white, black, and others (American Indian/AK Native, Asian/Pacific Islander). Marital status was divided into “married” and “single/other.” Surgery and radiotherapy were categorized into “Yes” and “No”. Distant (bone, brain, liver, and lung) metastases were divided into “Present” and “Absent.” The tumor stage was classified as local, regional, and distant. The T stage was assigned to T1, T2, and T3, while the N stage was assigned to N0 and N1. The primary site was identified as the following four categories: Appendicular (short and long bones of the lower and upper extremities); axial (pelvis and spine); rib, sternum, and clavicle; and other locations (skull, mandible, and other atypical locations).^16^

### 2.4 Statistical analysis

All data were analyzed using Microsoft Excel 2016 (Microsoft Corp.), SPSS (version 22.0), and R (version 4.0.3) software. A *p*‐value of less than 0.05 was considered statistically significant. The CSS was the primary endpoint of this study, defined as the time interval between the day of diagnosis and death caused by this tumor alone. The hazard ratios (HR) and corresponding 95% confidence interval (CI) were used to show the effect of enrolled variables on the CSS of those patients. First, each enrolled variable for this study was assigned a value for further statistical analysis (File S1). The statistical difference between the enrolled variables was identified using the Kaplan–Meier method and univariate Cox regression analysis. Then, variables with *p* < 0.05 were selected for multivariate Cox regression analysis to remove confounding effects and identify CSS‐related independent prognostic predictors for this subpopulation. A nomogram for predicting 1‐, 3‐, and 5‐year CSS in primary ES patients treated with chemotherapy was constructed based on CSS‐related independent prognostic predictors. At the same time, each CSS‐related independent prognostic predictor obtained a corresponding point in this CSS nomogram. Then, the nomogram's calibration and clinical benefit were verified by the 1‐, 3‐, and 5‐year calibration curves and corresponding decision curve analysis (DCA), respectively. Moreover, the nomogram's discrimination was evaluated using the corresponding time‐dependent area under the curve (AUC) values of 1‐, 3‐, and 5‐year receiver operating characteristic (ROC) curves. Besides, we plotted ROC curves for all independent prognostic factors to verify that the predictive validity of the constructed nomogram was better than a single independent prognostic factor. Furthermore, the patient's risk score was calculated using the above point assigned to the CSS‐related independent prognostic predictors, and the optimal cut‐off value for the risk score was determined based on the X‐tile software. Then, a risk stratification system was created to classify the mortality risk of that subpopulation into low‐, middle‐, and high‐risk subgroups. Finally, Kaplan–Meier method was generated to show the difference in CSS between the three subgroups.

## 3 RESULTS

### 3.1 Demographic and clinicopathologic characteristics

According to the inclusion and exclusion criteria, 393 primary ES patients treated with chemotherapy from the SEER database were enrolled in this study. They were randomly divided into a training cohort (*n* = 277, 70.00%) and a validation cohort (*n* = 116, 30.00%). Of these patients, 206 (52.42%) were aged between 13 and 28 years old, and 228 (58.02%) had a tumor size between 54‐ and 135‐mm. Patients' major sex and race were male (*n* = 233, 59.29%) and white (*n* = 344, 87.53%), respectively. The marital status of most patients was single/other (*n* = 356, 90.59%). Appendicular and axial were the top two primary sites, accounting for 79.64%. A small percentage of patients with T3 (*n* = 17, 4.33%) or N1(*n* = 36, 9.16%) stage. Besides, the disparity of tumor stage between localized, regional, and distant metastases was not apparent, and the percentage of patients with distant metastases involving lung, bone, liver, and brain were 17.30%, 12.21%, 0.76%, and 0.51%, respectively. As for the treatment, 63.61% received surgery, while the disparity between receiving radiotherapy and not receiving radiotherapy was not apparent (Table 1).

**TABLE 1:** The baseline demographic and clinicopathologic characteristics of the CSS‐related variables of primary ES patients treated with chemotherapy

Variables	Training cohort		Validation cohort		Total	
	277	70.00%	116	30.00%	393	100.00%
Age (years old)						
<13	94	33.94%	39	33.62%	133	33.84%
13–28	144	51.99%	62	53.45%	206	52.42%
<28	39	14.07%	15	12.93%	54	13.74%
Sex						
Male	163	58.84%	70	60.34%	233	59.29%
Female	114	41.16%	46	39.66%	160	40.71%
Race						
Black	10	3.61%	5	4.31%	15	3.82%
White	246	88.81%	98	84.48%	344	87.53%
Other	21	7.58%	13	11.21%	34	8.65%
Marital status						
Single/other	251	90.61%	105	90.52%	356	90.59%
Married	26	9.39%	11	9.48%	37	9.41%
Primary site						
Appendicular	135	48.74%	60	51.72%	195	49.62%
Axial	89	32.13%	29	25.00%	118	30.03%
Rib, sternum and clavicle	38	13.72%	14	12.07%	52	13.23%
Other locations	15	5.41%	13	11.21%	28	7.12%
Tumor size (mm)						
<54	64	23.11%	29	25.00%	93	23.66%
54–135	162	58.48%	66	56.90%	228	58.02%
>135	51	18.41%	21	18.10%	72	18.32%
SEER historic stage A						
Localized	90	32.49%	37	31.90%	127	32.32%
Regional	107	38.63%	36	31.03%	143	36.39%
Distant	80	28.88%	43	37.07%	123	31.29%
Derived AJCC T						
T1	130	46.93%	60	51.72%	190	48.35%
T2	135	48.74%	51	43.97%	186	47.33%
T3	12	4.33%	5	4.31%	17	4.32%
Derived AJCC N						
N0	257	92.78%	100	86.21%	357	90.84%
N1	20	7.22%	16	13.79%	36	9.16%
Bone metastasis						
Absent	247	89.17%	98	84.48%	345	87.79%
Present	30	10.83%	18	15.52%	48	12.21%
Brain metastasis						
Absent	275	99.28%	116	100.00%	391	99.49%
Present	2	0.72%	0	0.00%	2	0.51%
Lung metastasis						
Absent	231	83.39%	94	81.03%	325	82.70%
Present	46	16.61%	22	18.97%	68	17.30%
Liver metastasis						
Absent	275	99.28%	115	99.14%	390	99.24%
Present	2	0.72%	1	0.86%	3	0.76%
Radiotherapy						
No	141	50.90%	59	50.86%	200	50.89%
Yes	136	49.10%	57	49.14%	193	49.11%
Surgery						
No	103	37.18%	40	34.48%	143	36.39%
Yes	174	62.82%	76	65.52%	250	63.61%

Abbreviation: CSS, cancer specific survival.

### 3.2 Identification of the independent prognostic predictors for CSS in primary ES patients treated with chemotherapy

**FIGURE 2:**
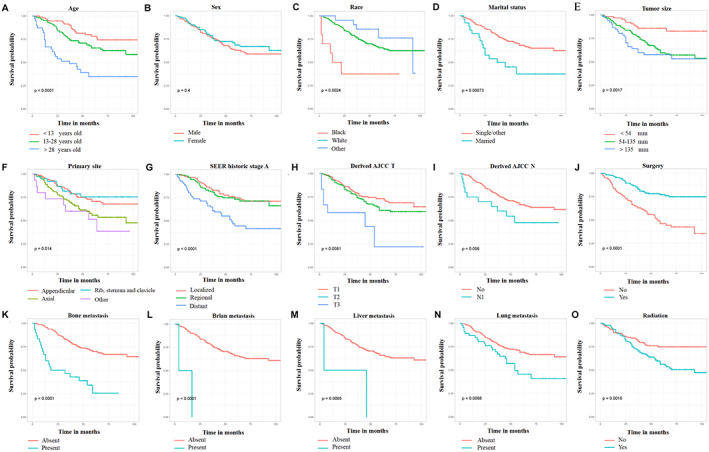
Kaplan–Meier survival curves were performed on cancer‐specific survival (CSS)‐related risk variables in primary Ewing sarcoma (ES) patients treated with chemotherapy. (A) age, (B) sex, (C) race, (D) marital status, (E) tumor size, (F) primary site, (G) tumor stage, (H) T stage, (I) N stage, (J) surgery, (K) bone metastasis, (L) brain metastasis, (M) liver metastasis, (N) lung metastasis, and (O) radiation.

Univariate Cox regression analysis and Kaplan–Meier method identified that age, race, marital status, primary site, tumor stage, tumor size, T stage, distant (lung, bone, liver, and brain) metastases, surgery, and radiotherapy were identified as risk‐related variables for CSS (*p* < 0.05, Figure 2). Then, those above CSS‐related variables were used to perform a multivariate Cox regression analysis to eliminate the effects of confounding variables. The result showed that age, tumor size, bone metastasis, and surgery were CSS‐related independent prognostic predictors in this subpopulation. As shown in Table 2, older age, tumor size over 135 mm, bone metastasis, and not receiving surgery were all related to higher mortality risk (Table 2).

**TABLE 2:** The univariate and multivariate Cox regression analyses of the CSS‐related variables of primary ES patients treated with chemotherapy

Variables	Univariate analysis		Multivariate analysis	
	OR (95% CI)	*p* Value	OR (95% CI)	*p* Value
Age (years old)				
<13	Reference		Reference	
13–28	1.705 (1.013–2.868)	0.044	1.200 (0.704–2.047)	0.503
>28	4.510 (2.485–8.184)	<0.001	4.358 (2.379–7.983)	<0.001
Sex				
Male	Reference			
Female	0.838 (0.551–1.272)	0.406		
Race				
Black	Reference			
White	0.274 (0.119–0.628)	0.002		
Other	0.183 (0.056–0.600)	0.005		
Marital status				
Single/other	Reference			
Married	2.510 (1.441–4.369)	0.001		
Primary site				
Appendicular	Reference			
Axial	1.702 (1.085–2.669)	0.021		
Rib, sternum and clavicle	0.834 (0.403–1.725)	0.625		
Other locations	2.390 (1.115–5.126)	0.025		
Tumor size (mm)				
<54	Reference		Reference	
54–135	2.766 (1.417–5.399)	0.003	2.731 (1.365–5.463)	0.005
>135	3.556 (1.683–7.514)	<0.001	4.084 (1.872–8.908)	<0.001
SEER historic stage A				
Localized	Reference			
Regional	1.118 (0.646–1.933)	0.690		
Distant	2.614 (1.566–4.362)	<0.001		
Derived AJCC T				
T1	Reference			
T2	1.316 (0.859–2.017)	0.207		
T3	3.369 (1.498–7.574)	0.003		
Derived AJCC N				
N0	Reference			
N1	1.871 (0.971–3.607)	0.061		
Bone metastasis				
Absent	Reference		Reference	
Present	3.930 (2.385–6.476)	<0.001	3.002 (1.741–5.177)	<0.001
Brain metastasis				
Absent	Reference			
Present	16.247 (3.848–68.603)	<0.001		
Lung metastasis				
Absent	Reference			
Present	1.893 (1.181–3.034)	0.008		
Liver metastasis				
Absent	Reference			
Present	5.599 (1.374–22.814)	0.016		
Radiotherapy				
No	Reference			
Yes	1.955 (1.278–2.991)	0.002		
Surgery				
No	Reference		Reference	
Yes	0.351 (0.232–0.530)	<0.001	0.490 (0.315–0.763)	0.002

Abbreviation: CSS, cancer specific survival.

### 3.3 Establishment and verification of the prognostic nomogram for CSS in primary ES patients treated with chemotherapy

**FIGURE 3:**
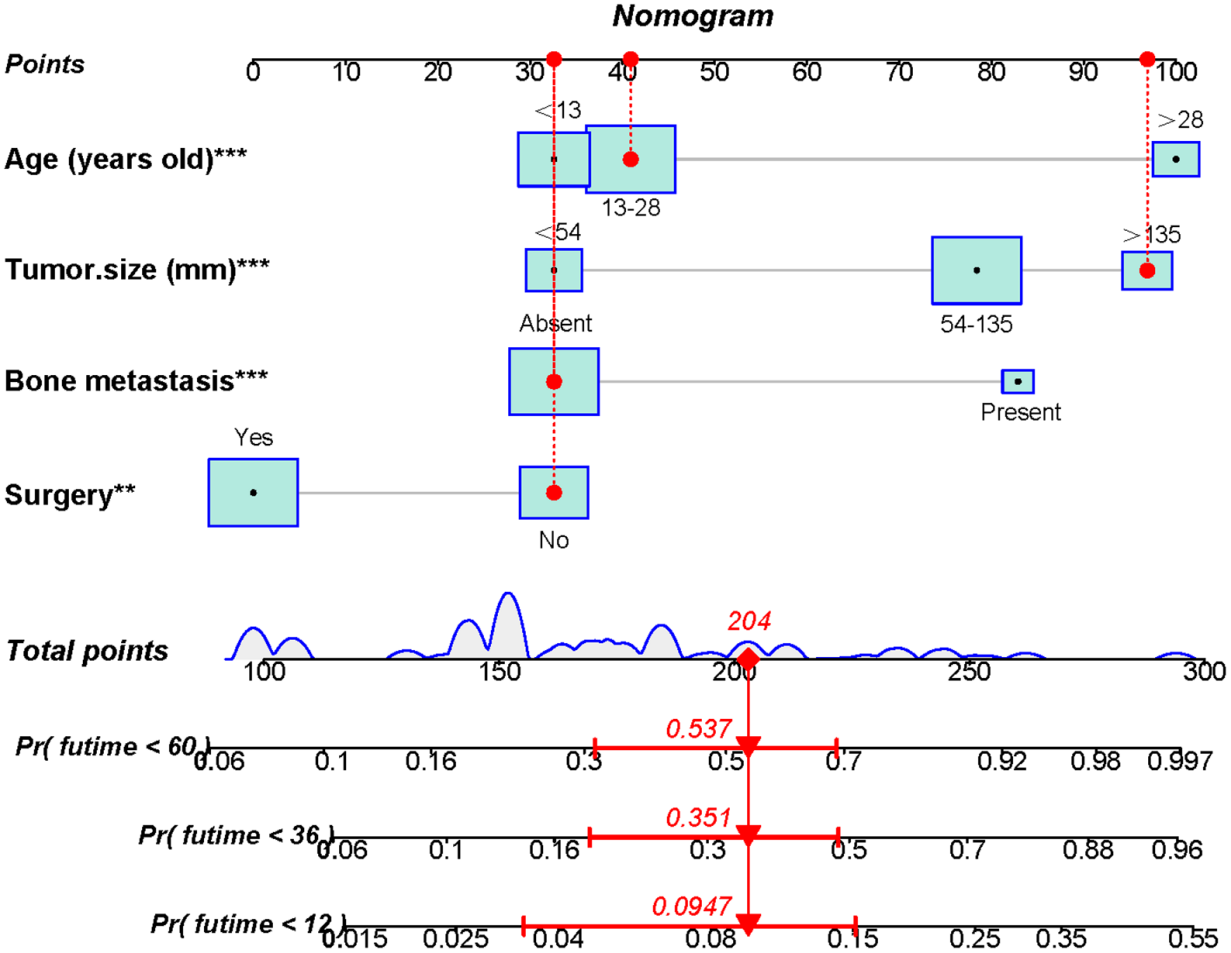
The prognostic nomogram predicts the 1‐, 3‐ and 5‐year CSS in primary ES patients treated with chemotherapy. Specifically, when such a patient consults about his CSS, we can sum the scores of the obtained independent prognostic predictors to get a total score and draw a vertical line from the total score to the bottom timeline. For example, the primary tumor, a 25 years old male patient with a 150 mm diameter ES, had received chemotherapy with no surgical treatment and no bone metastasis. The corresponding total score of he is 41 (25 years old) + 97 (150 mm of tumor size) + 33 (no bone metastasis) + 33 (no surgery) = 204, and the corresponding death risk possible at 1‐, 3‐, and 5‐ year are 0.0947, 0.351, and 0.537, respectively, while the corresponding CSS of the patient at 1‐, 3‐, and 5‐ year are 0.9053, 0.649, and 0.463, respectively.

**FIGURE 4:**
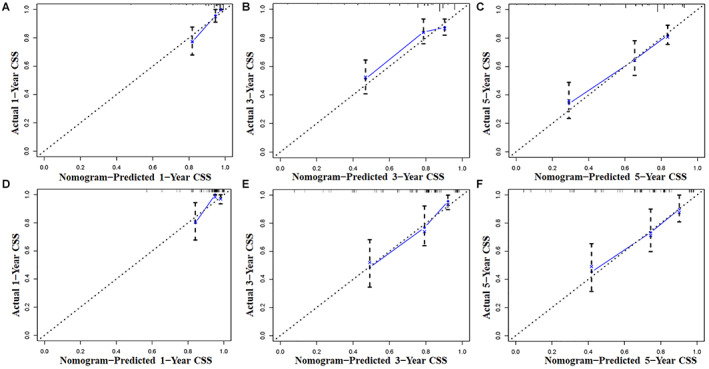
The calibration curves of the nomogram were used to predict CSS in primary ES patients treated with chemotherapy at 1‐(A), 3‐(B), and 5‐(C) year in the training cohort and 1‐(D), 3‐(E), and 5‐(F) year in the validation cohort, respectively.

**FIGURE 5:**
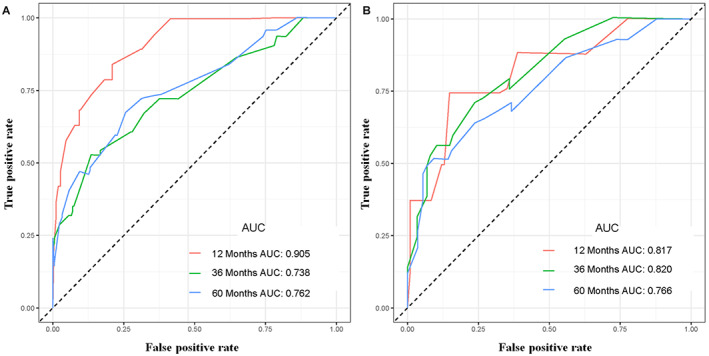
The 1‐, 3‐, and 5‐year receiver operating characteristic curves of primary ES patients treated with chemotherapy in the training (A) and validation (B) cohorts.

**FIGURE 6:**
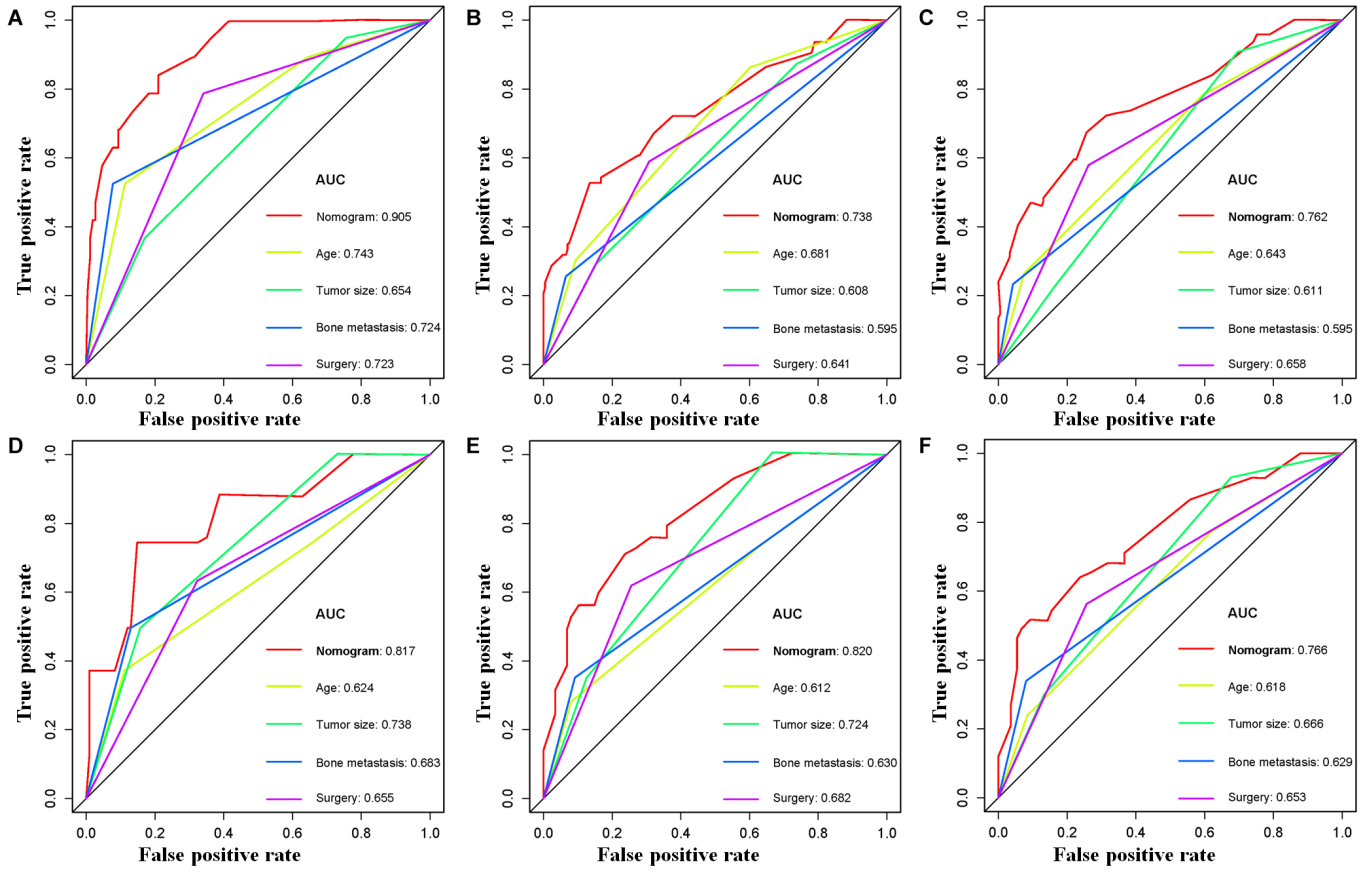
Comparison of prediction accuracy between the constructed novel nomogram and each CSS‐related independent prognostic predictor in primary ES patients treated with chemotherapy at 1‐(A), 3‐(B), and 5‐(C) year in the training cohort and 1‐(D), 3‐(E), and 5‐(F) year in the validation cohort, respectively.

**FIGURE 7:**
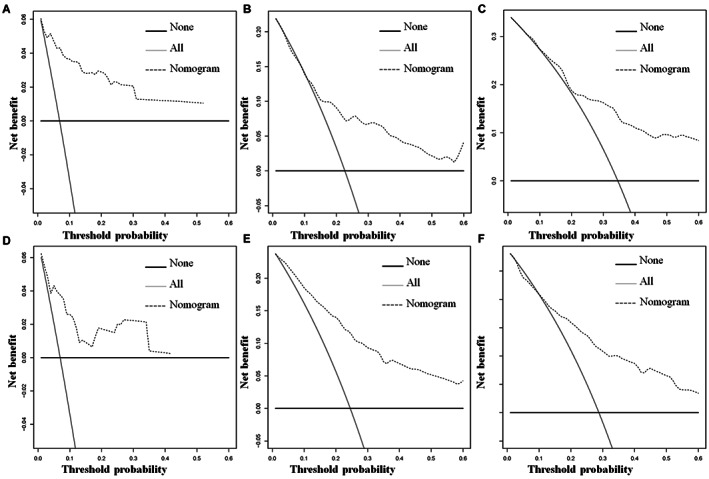
The decision curve analysis of the constructed novel nomogram was used to predict the 1‐(A), 3‐(B), and 5‐(C) year CSS in the training cohort, and the 1‐(D), 3‐(E), and 5‐(F) year CSS in the validation cohort for primary ES patients treated with chemotherapy.

Using a quantitative method, the four aforementioned CSS‐related independent prognostic predictors were gathered to construct a prognostic nomogram for predicting 1‐, 3‐, and 5‐year CSS in this subpopulation. The points for each independent prognostic predictor were summed to obtain the total point for the predicted individual (Table 3). The patient's 1‐, 3‐, and 5‐year mortality rates can be calculated by drawing a vertical line from the total score row to the bottom timeline, and the corresponding CSS can be obtained (Figure 3). As revealed in Figure 4, the 1‐, 3‐, and 5‐year calibration curves showed good agreement between actual and predicted outcomes based on the constructed nomogram using bootstrap resampling 1000 times (Figure 4). The 1‐, 3‐, and 5‐year AUCs for CSS in the training cohort were 0.905, 0.738, and 0.762, respectively, and those in the validation cohort were 0.817, 0.820, and 0.766, indicating that the constructed nomogram possessed a good discriminatory power (Figure 5). Then, in order to demonstrate that the constructed nomogram had the best predictive power, we also compared the predictive accuracy of each CSS‐related independent prognostic predictor with the constructed nomogram (Figure 6). The results showed that the 1‐, 3‐, and 5‐year AUCs of each CSS‐related independent prognostic predictor were significantly lower than that of the constructed nomogram in both cohorts, indicating that the prediction accuracy of the nomogram was better than each CSS‐related independent prognostic predictor. Moreover, DCA showed that the constructed nomogram had an excellent positive net benefit over a wide range of mortality risks, suggesting that the nomogram had a high value of practical clinical application (Figure 7).

**TABLE 3:** The detailed point of each independent prognostic factor in the CSS nomogram

CSS‐related independent variables	Corresponding point assignments in CSS nomogram
Age (years old)	
<13	33
13–28	41
>28	100
Tumor size (mm)	
<54	33
54–135	78
>135	97
Bone metastasis	
Absent	33
Present	83
Surgery	
No	33
Yes	0

Abbreviation: CSS, cancer specific survival.

### 3.4 Risk stratification system for CSS

**FIGURE 8:**
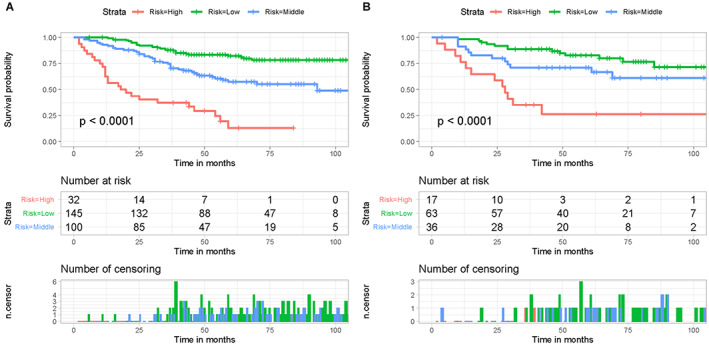
Kaplan–Meier survival analysis and log‐rank tests were performed to compare CSS of primary ES patients treated with chemotherapy in the three risk subgroups in the training cohort (A) and validation cohort (B). The high‐risk subgroup had a worse prognosis than those in the low‐risk subgroup.

In addition to predicting patients' CSS, it is essential to categorize patients based on their mortality risk for individualized management. Therefore, to further verify the stability and performance of the nomogram from different dimensions, a cancer unique mortality risk stratification system was constructed based on the four aforementioned CSS‐related independent prognostic predictors. Specifically, patients' total points were calculated. According to X‐tile software, the optimal cut‐off values for the total point were 166 and 230 (File S1). Thus, patients were further divided into three mortality risk subgroups: low‐ (<166), middle‐ (166–230), and high‐ (>230), and Kaplan–Meier method for three mortality risk subgroups was drawn meanwhile (Figure 8). As shown in Figure 8, the risk stratification system could effectively categorize patients in both cohorts into three subgroups with significant differences (*p* < 0.05). Patients with high mortality risk had a poor CSS than those with low mortality risk, indicating that the cancer unique mortality risk stratification system constructed from the prognostic nomogram had an excellent predictive ability and realized better cancer patient management.

## 4 DISCUSSION

ES is the second most common malignant bone tumor in childhood and adolescence, with the highest incidence in the second decade of life and a certain male predominance.^17^ ES is composed of small round cells that express high levels of CD99.^18^, ^19^ White ethnicity is more affected than Asian and black ethnicities.^3^ ES predominantly occurs in the pelvis, femur, tibia, and ribs and mainly affects children, adolescents, and young adults.^20^ With the promotion of neoadjuvant chemotherapy in ES, the 5‐year OS of ES patients has improved to 60%. However, the relatively low survival rate still limits the better treatment benefits for patients and cannot meet the expectations of the patient that underwent chemotherapy.

Personalized medicine is becoming increasingly important in cancer treatment to avoid under‐treatment of high mortality risk patients, over‐treatment of low mortality risk patients, or those with limited expected benefit from treatment. The development of prognostic survival models could help stratify treatment according to the mortality risk of individual patients, allowing risk‐ and response‐adapted treatment strategies to be developed to maximize patient benefit. However, fewer studies have focused on discovering independent prognostic predictors related to the survival of primary ES patients treated with chemotherapy. Therefore, this study sought to identify independent prognostic predictors associated with CSS of this subpopulation.

Our findings suggest that age, tumor size, bone metastasis, and surgery were independent prognostic predictors for CSS in this subpopulation. Then, we developed the first nomogram for predicting the 1‐, 3‐, and 5‐ year CSS of this subpopulation. The constructed nomogram showed good consistency between predictions and actual observations. In addition, DCA showed that the constructed nomogram had a high value in practical clinical application. We also used this nomogram to develop a mortality risk stratification system that categorizes those patients into three risk subgroups: high, middle, and low, enabling clinicians to achieve patient risk‐stratified management and targeted treatment.

Age has been identified as an essential survival prognostic predictor for OS and CSS in ES patients, although the exact mechanism remains unclear.^21^ Our study found that with increasing age, the CSS of primary ES patients treated with chemotherapy decreased, and elderly patients (>28 years old) had the worst prognosis compared to younger ones. The pediatric immune system has a natural tumor suppressor effect, which can inhibit tumor growth to a certain extent and delay the probability of distant metastases.^22^, ^23^ Alternatively, Baldini et al. proposed that adults might not have enough bone marrow reserve to tolerate the prolonged chemotherapy regimens that had produced good results for children,^21^ which was also a possibility for a poorer prognosis. In addition, younger patients are in relatively good health and could tolerate surgery, chemotherapy, and radiotherapy. Thus, it could ensure patients receive an efficient course and dose of treatment and fewer post‐operative and other treatment complications than older people, thus having a longer survival time.

Our study showed that tumor size was an independent prognostic predictor for CSS, consistent with previous studies.^5^, ^6^ Results showed that larger the tumor size, worse the patient's prognosis. The reason may be that it is easier for larger tumor to compress the surrounding normal tissues, such as nerves and blood vessels, and even cause pathological fractures and other symptoms after a long time, which will adversely affect the patient's quality of life. Moreover, the primary purpose of surgery for ES is to obtain a negative margin. For ES located in the pelvis, limited by particular anatomical factors, the larger the tumor, the more challenging it is to achieve negative margins. Therefore, previous literature also reported that ES located in the pelvis had a poor prognosis, and the survival time of those patients was further reduced.^24^, ^25^

The most common distant metastatic sites of primary ES are lung (36.5–49.1%) and bone (34.6–38.5%), followed by liver and brain, which account for a small proportion.^4^, ^26^, ^27^, ^28^, ^29^ In our study, the frequency of metastases in this subpopulation was lung (17.30%), bone (12.21%), liver (0.76%), and brain (0.51%), which was consistent with the frequency reported in previous literature, but the ratio has dropped significantly. Since chemotherapy was mainly effective control for distant metastases, it is of great value in treating primary ES. It should be used as an essential part of treatment, even for those patients who do not have distant metastases at the time of presentation. However, not all sites of distant metastases were independently associated with patient survival. Several studies have found that ES associated with adjacent or distant bone/bone marrow metastases predicted a poor survival compared with lung metastasis.^30^ Primary ES patients with distant metastases at diagnosis typically had a significantly reduced OS of less than 30%, whereas patients with solitary lung metastasis had a better OS of approximately 50%.^20^ Paulussen et al. showed that ES with solitary lung and bone metastases had an event‐free survival (EFS) of 0.34 and 0.28, respectively.^29^ A study of patients with primary disseminated multifocal ES showed that survival was better for ES with lung metastasis than bone.^31^ Zhang et al. also showed that only bone metastasis was associated with poorer survival in this subpopulation.^4^ Our study reached the same conclusion. The possible reason for this phenomenon is that whole lung radiotherapy (WLR) could effectively treat lung metastasis. Elghazawy et al. and Bölling et al. both showed that WLR improved 5‐year EFS and 5‐year pulmonary relapse‐free survival with acceptable toxicity, significantly improving long‐term outcomes.^32^, ^33^ Furthermore, Paulussen et al. proposed that tumor cells that ‘nest’ to bone/bone marrow had higher malignant potential than those merely settling in the first capillary bed after being detached from the primary tumor.^29^ This phenomenon has important implications for treating organ‐specific metastases and for the prognosis of primary metastatic ES.

Surgery is the most effective treatment for ES. Before applying chemotherapy, the primary surgical option for ES was amputation. With the development of chemotherapy, limb‐sparing surgery has gained attention in the treatment of ES. Surgery is usually performed on lower extremity lesions in ES patients whose epiphysis has not yet closed, those prone to pathological fractures, and non‐weight‐bearing bones, such as ribs, clavicles, and fibula. The goal of surgery is to completely remove the tumor with negative margins, including 2–3 cm of normal tissue. In the past, it was considered that the indications for surgical treatment were that the surgery would not result in severe functional impairment and no particular reconstruction was required afterward. However, it is now considered that surgery should be performed for all tumors that can be removed entirely. Lee et al. showed that patients treated with local surgery had a better OS than those who did not. They also analyzed the impact of limb‐salvage surgery and amputation on survival. The results showed that patients who received local or radical resection and limb‐salvage surgery had a significantly lower risk of death. Moreover, compared with amputation, limb salvage surgery could significantly improve patients' daily function and ensure a better quality of life and more prolonged survival.^34^ Verma et al. compared the benefits of surgery in adults and children with ES, and the results showed that surgery significantly improved OS in both adults and children. However, the benefits of surgery in children were more significant than in adults,^35^ which was similar to our findings.

ES is extremely sensitive to radiotherapy. The tumor could shrink rapidly, and local pain could be significantly reduced or disappear with low‐dose irradiation. However, after our study's multivariate Cox regression analysis, radiotherapy was not an independent variable associated with CSS in this subpopulation. Many previous studies have also concluded that radiotherapy is not independently associated with OS or CSS.^5^, ^36^ Radiotherapy could damage the epiphyseal cartilage, blood vessels, nerves, and soft tissues, leading to osteodystrophy, deformity, fibrosis, or pathological fractures, resulting in severe limb dysfunction, affecting patients' life quality. Therefore, radiotherapy alone cannot always be an effective local control method. At the same time, the high recurrence rate caused by radiotherapy alone was also an essential factor leading to the failure of long‐term treatment.^37^ Stahl et al. showed that the 5‐year survival rate of ES patients who relapse within 24 months after diagnosis was less than 10%.^7^

Inevitably, this study has some limitations. First, retrospective studies inevitably suffer from selection bias. Second, the specific chemotherapy regimens used to treat this subpopulation, the adverse effects of chemotherapy and countermeasures, and the radiotherapy doses and detailed surgical plans were not recorded in the SEER database. Third, the performance and reliability of the nomogram need to be verified in other medical centers or databases. Future studies could add tumor markers and molecular biological variables to develop a more comprehensive nomogram to predict CSS in primary ES patients treated with chemotherapy.

## 5 CONCLUSIONS

We have constructed and validated a novel prognostic nomogram to predict CSS of primary ES patients treated with chemotherapy. The nomogram could facilitate the clinical application of the prediction model and be used as a supplementary predictive tool in clinical practice for treatment decision‐making, surveillance, counseling, and patient management.

## AUTHOR CONTRIBUTIONS

**Chao Huang:** Conceptualization (equal); data curation (equal); formal analysis (equal); methodology (equal); resources (equal); writing – original draft (equal). **Qiu‐Ping Yu:** Conceptualization (equal); data curation (equal); investigation (equal); writing – original draft (equal). **Zichuan Ding:** Data curation (equal); investigation (equal); resources (equal); software (equal). **Zongke Zhou:** Project administration (equal); supervision (equal); visualization (equal); writing – review and editing (equal). **Xiaojun Shi:** Funding acquisition (lead); project administration (lead); supervision (lead); validation (lead); visualization (lead); writing – review and editing (lead). All authors read and approved the final manuscript.

## FUNDING INFORMATION

This research was supported by the National Natural Science Foundation of China (No. 81871780, 82072420).

## CONFLICT OF INTEREST

The authors declare no competing interests.

## ETHICS APPROVAL

All methods were carried out in accordance with relevant guidelines and regulations. Data extraction and usage has been approved by SEER Program. All the data can be found in the SEER dataset: https://seer.cancer.gov/seerstat/. We obtained access to the SEER database after obtaining permission to access research data files with the reference number 16336‐Nov2020.

## INFORMED CONSENT

All data collected in this study have consent for publication.

## Supporting information


Figure S1–S3

Table S1

## Data Availability

The dataset from the SEER database that was generated and/or analyzed during the current study is available in the SEER dataset repository (https://seer.cancer.gov/). The datasets generated during and/or analyzed during the current study are available from the corresponding author on reasonable request.
