# Supplementary material for: The clinical characteristics, novel predictive tool, and risk classification system for primary Ewing sarcoma patients that underwent chemotherapy: A large population‐based retrospective cohort study
Source: Cancer Med. 2022 Oct 21;12(5):6244–59. doi: 10.1002/cam4.5379 (PMC10028057; doi:10.1002/cam4.5379)
Supplement: Supplementary file 1 — Figure S1–S3 [file CAM4-12-6244-s001.docx]

**Supplementary Figures**


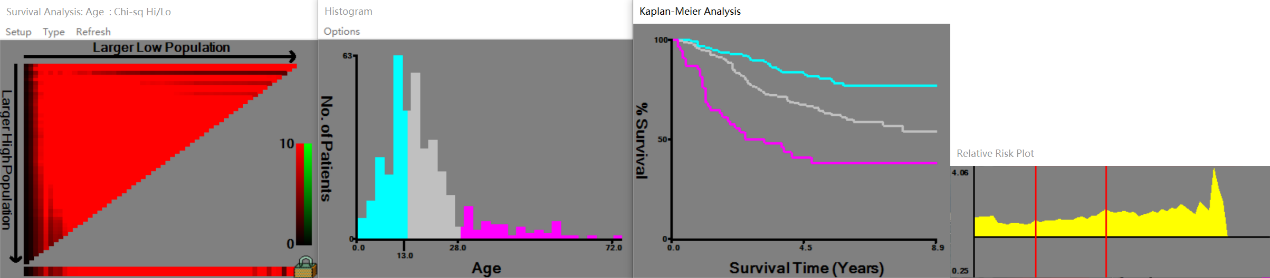


**Supplementary Figure 1:** According to the X-tile software, the optimal cut-off values for the age were determined to be 13 and 28 (years old).


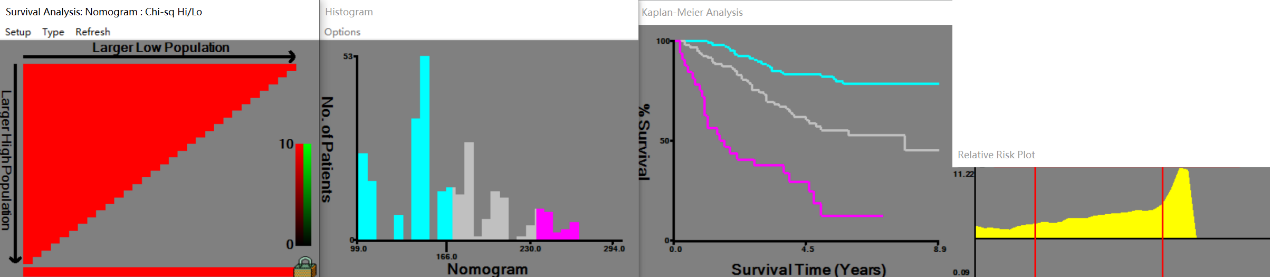


**Supplementary Figure 2:** According to the X-tile software, the optimal cut-off values for the tumor size were determined to be 54 and 135 (mm).


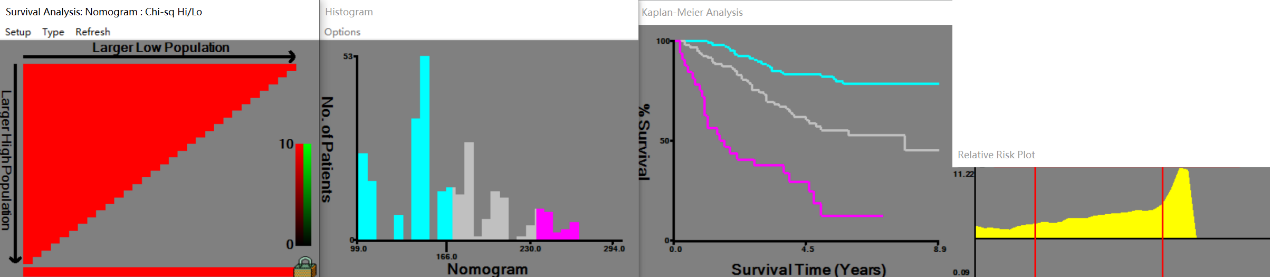


**Supplementary Figure 3:** According to the X-tile software, the optimal cut-off values for the cancer-specific mortality score were determined to be 166 and 230.
