# Supplementary material for: The clinical characteristics, novel predictive tool, and risk classification system for primary Ewing sarcoma patients that underwent chemotherapy: A large population‐based retrospective cohort study
Source: Cancer Med. 2022 Oct 21;12(5):6244–59. doi: 10.1002/cam4.5379 (PMC10028057; doi:10.1002/cam4.5379)
Supplement: Supplementary file 2 — Table S1 [file CAM4-12-6244-s002.docx]

**Table S1.** The values assigned ​​to CSS-related variables in this study.

| **CSS-related variables** | **Assigned values** |
| --- | --- |
| **Age (years old)** | |
| ＜13 | 0 |
| 13-28 | 1 |
| >28 | 2 |
| **Race** | |
| Black | 1 |
| White | 2 |
| Other | 3 |
| **Marital status** | |
| Single/other | 0 |
| Married | 1 |
| **Primary site** | |
| Appendicular | 1 |
| Axial | 2 |
| Rib, sternum and clavicle | 3 |
| Other locations | 4 |
| **Tumor grade** | |
| Ⅰ | 1 |
| Ⅱ | 2 |
| Ⅲ | 3 |
| Ⅳ | 4 |
| **SEER historic stage A** | |
| Localized | 1 |
| Regional | 2 |
| Distant | 3 |
| **Tumor size (mm)** | |
| <54 | 1 |
| 54-135 | 2 |
| >135 | 3 |
| **Derived AJCC T** | |
| T1 | 1 |
| T2 | 2 |
| T3 | 3 |
| **Derived AJCC N** | |
| N0 | 0 |
| N1 | 1 |
| **Bone metastasis** | |
| Absent | 0 |
| Present | 1 |
| **Brain metastasis** | |
| Absent | 0 |
| Present | 1 |
| **Lung metastasis** | |
| Absent | 0 |
| Present | 1 |
| **Liver metastasis** | |
| Absent | 0 |
| Present | 1 |
| **Radiotherapy** | |
| No | 0 |
| Yes | 1 |
| **Surgery** | |
| No | 0 |
| Yes | 1 |

CSS: cancer specific survival
